# Supplementary material for: Upper-plate conduits linked to plate boundary that hosts slow earthquakes
Source: Nat Commun. 2023 Sep 20;14:5101. doi: 10.1038/s41467-023-40762-4 (PMC10511545; doi:10.1038/s41467-023-40762-4)
Supplement: Supplementary file 1 — Supplementary Information File [file 41467_2023_40762_MOESM1_ESM.pdf]

Supplementary Information

Upper-plate conduits linked to plate boundary that hosts slow earthquakes

Ryuta Arai[1*], Seiichi Miura[1], Yasuyuki Nakamura[1], Gou Fujie[1], Shuichi Kodaira[1], Yuka Kaiho[1], Kimihiro Mochizuki[2], Rie Nakata[2], Masataka Kinoshita[2], Yoshitaka Hashimoto[3], Yohei Hamada[4], Kyoko Okino[5]

[1]Research Institute for Marine Geodynamics, Japan Agency for Marine-Earth Science and Technology, 3173-25 Showa-machi, Kanazawa-ku, Yokohama, Kanagawa 236-0001, Japan
[2]Earthquake Research Institute, The University of Tokyo, 1-1-1 Yayoi Bunkyo-ku, Tokyo 113-0032, Japan
[3]Faculty of Science and Technology, Kochi University, Akebonocho 2-5-1, Kochi 780-8520, Japan
[4]Kochi Institute for Core Sample Research, Japan Agency for Marine-Earth Science and Technology, 200 Monobe Otsu Nankoku, Kochi 783-8502, Japan
[5]Atmosphere and Ocean Research Institute, The University of Tokyo, 5-1-5 Kashiwanoha Kashiwa, Chiba 277-8564, Japan

[*]**Corresponding author:** Email- ryuta@jamstec.go.jp

This supporting information file contains 11 supplementary figures and 1 supplementary table that provides detailed descriptions on seismic data and velocity modeling.

Figure list:

Supplementary Figure 1. Comparison of ocean bottom seismograph (OBS) records of S049 with synthetic waveforms.
Supplementary Figure 2. Comparison of ocean bottom seismograph (OBS) records of S045 with synthetic waveforms.
Supplementary Figure 3. Comparison of ocean bottom seismograph (OBS) records of S038 with synthetic waveforms.
Supplementary Figure 4. Comparison of ocean bottom seismograph (OBS) records of S033 with synthetic waveforms.
Supplementary Figure 5. Comparison of ocean bottom seismograph (OBS) records of S029 with synthetic waveforms.
Supplementary Figure 6. Comparison of ocean bottom seismograph (OBS) records of S016 with synthetic waveforms.
Supplementary Figure 7. Comparison of ocean bottom seismograph (OBS) records of S005 with synthetic waveforms
Supplementary Figure 8. Comparison of ocean bottom seismograph (OBS) records of S001 with synthetic waveforms.
Supplementary Figure 9. Initial and final Vp modeling results.
Supplementary Figure 10. Checkerboard resolution tests for first-arrival traveltime tomography (FAT) and full waveform inversion (FWI) tomography.
Supplementary Figure 11. Offset and time damping used in FWI analysis.

Table List:

Supplementary Table 1. FWI parameters.

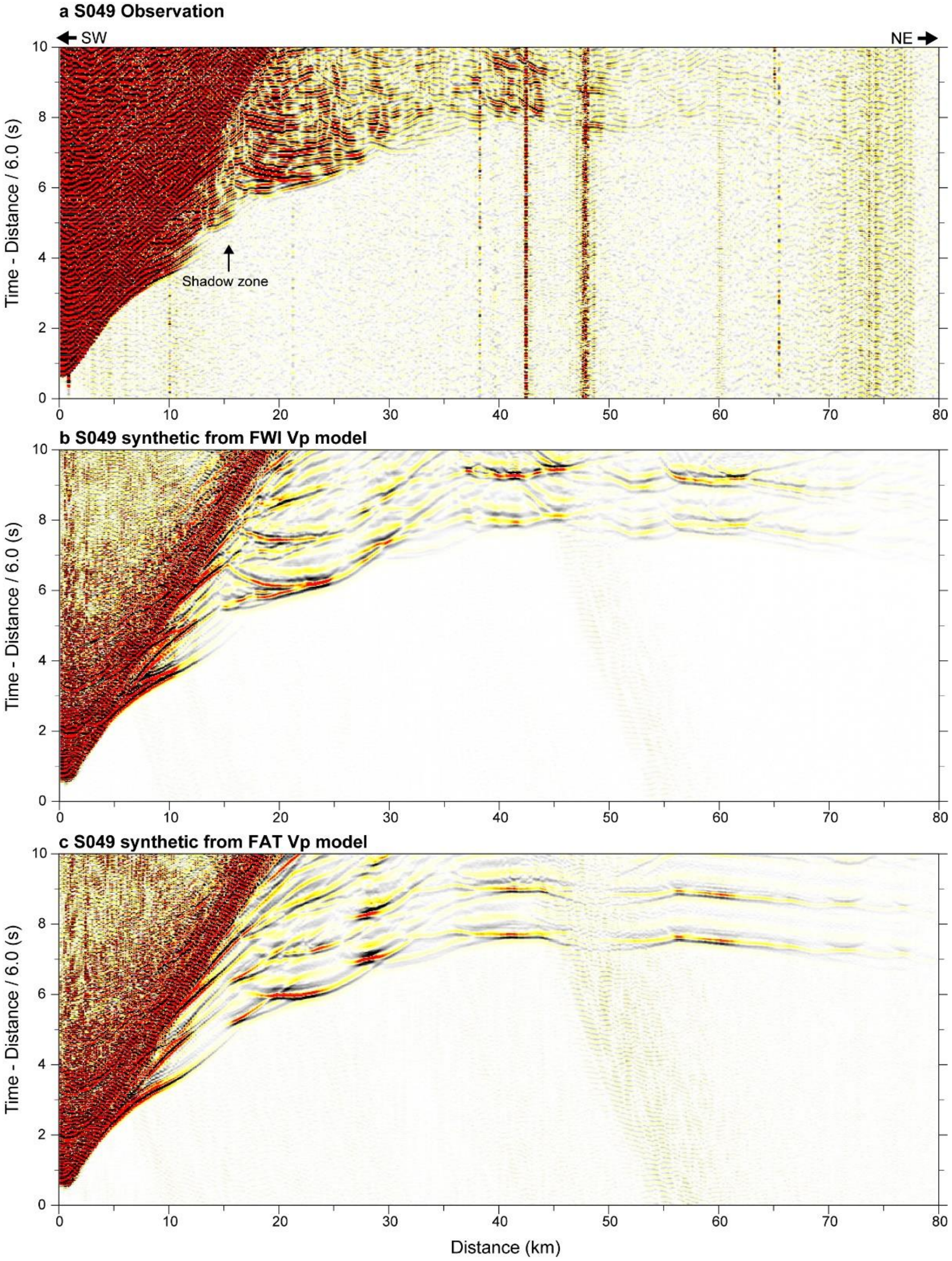

Supplementary Figure 1. Comparison of ocean bottom seismograph (OBS) records of S049 with synthetic waveforms. (a) Observed records of vertical geophone data. (b) Synthetic waveforms calculated from the final full waveform inversion (FWI) P-wave velocity (Vp) model (Supplementary Fig. 9c). (c) Synthetic waveforms calculated from the first-arrival traveltime tomography (FAT) Vp model (Supplementary Fig. 9b); we applied a 2–8-Hz band-pass filter.

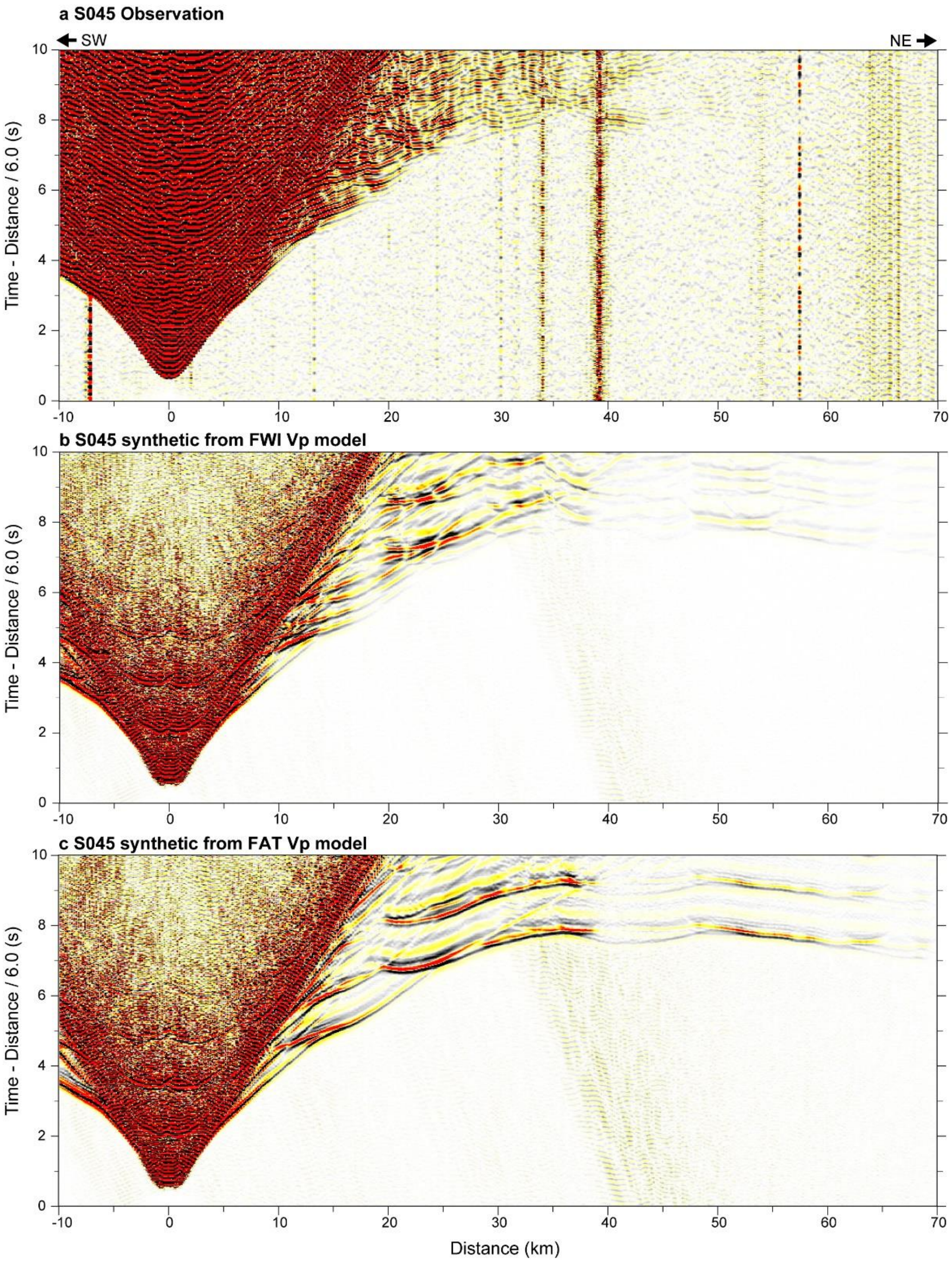

Supplementary Figure 2. Comparison of ocean bottom seismograph (OBS) records of S045 with synthetic waveforms. (a) Observed records of vertical geophone data. (b) Synthetic waveforms calculated from the final full waveform inversion (FWI) P-wave velocity (Vp) model (Supplementary Fig. 9c). (c) Synthetic waveforms calculated from the first-arrival traveltime tomography (FAT) Vp model (Supplementary Fig. 9b); we applied a 2–8-Hz band-pass filter.

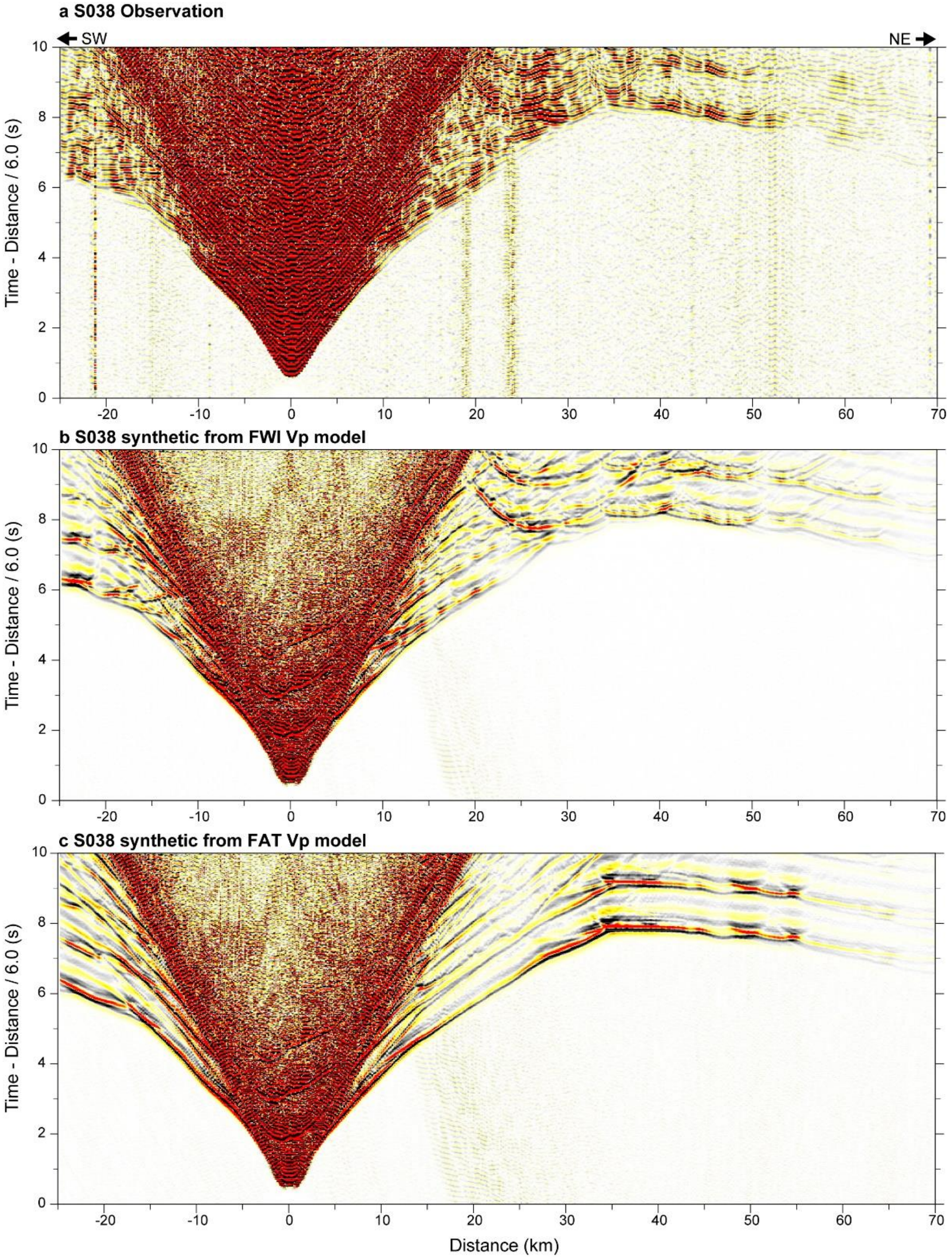

Supplementary Figure 3. Comparison of ocean bottom seismograph (OBS) records of S038 with synthetic waveforms. (a) Observed records of vertical geophone data. (b) Synthetic waveforms calculated from the final full waveform inversion (FWI) P-wave velocity (Vp) model (Supplementary Fig. 9c). (c) Synthetic waveforms calculated from the first-arrival traveltime tomography (FAT) Vp model (Supplementary Fig. 9b); we applied a 2–8-Hz band-pass filter.

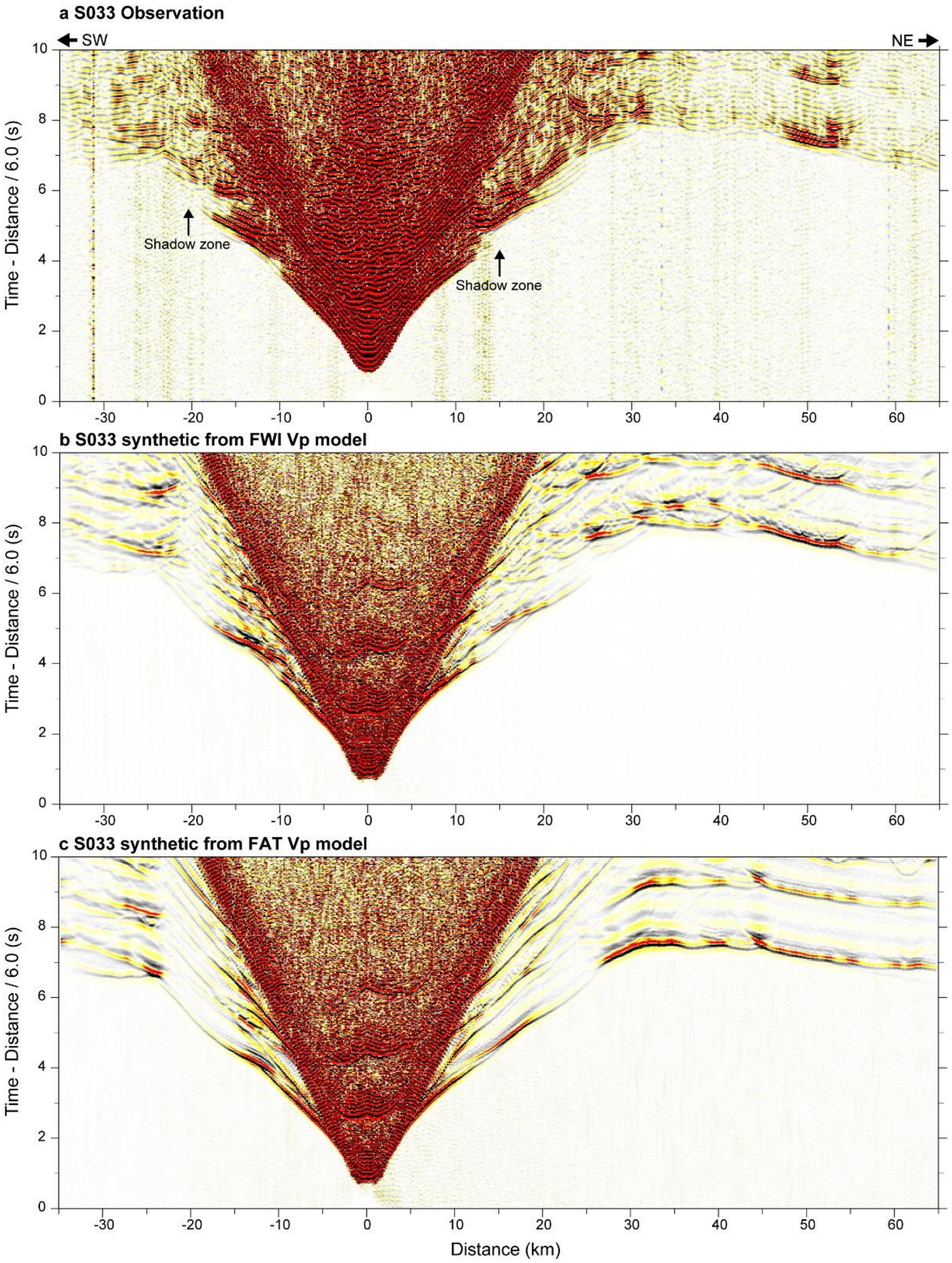

Supplementary Figure 4. Comparison of ocean bottom seismograph (OBS) records of S033 with synthetic waveforms. (a) Observed records of vertical geophone data. (b) Synthetic waveforms calculated from the final full waveform inversion (FWI) P-wave velocity (Vp) model (Supplementary Fig. 9c). (c) Synthetic waveforms calculated from the first-arrival traveltime tomography (FAT) Vp model (Supplementary Fig. 9b); we applied a 2–8-Hz band-pass filter.

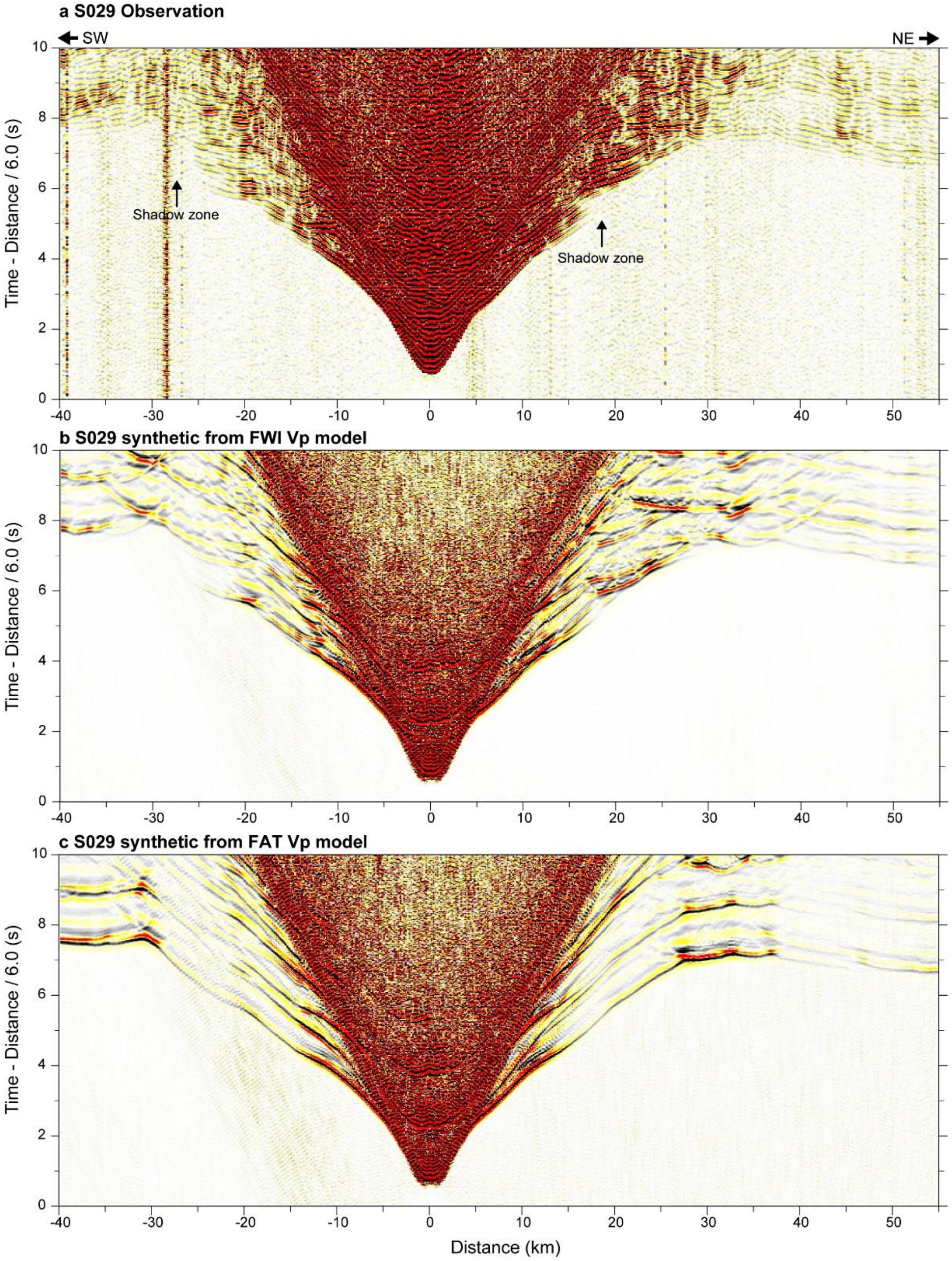

Supplementary Figure 5. Comparison of ocean bottom seismograph (OBS) records of S029 with synthetic waveforms. (a) Observed records of vertical geophone data. (b) Synthetic waveforms calculated from the final full waveform inversion (FWI) P-wave velocity (Vp) model (Supplementary Fig. 9c). (c) Synthetic waveforms calculated from the first-arrival traveltime tomography (FAT) Vp model (Supplementary Fig. 9b); we applied a 2–8-Hz band-pass filter.

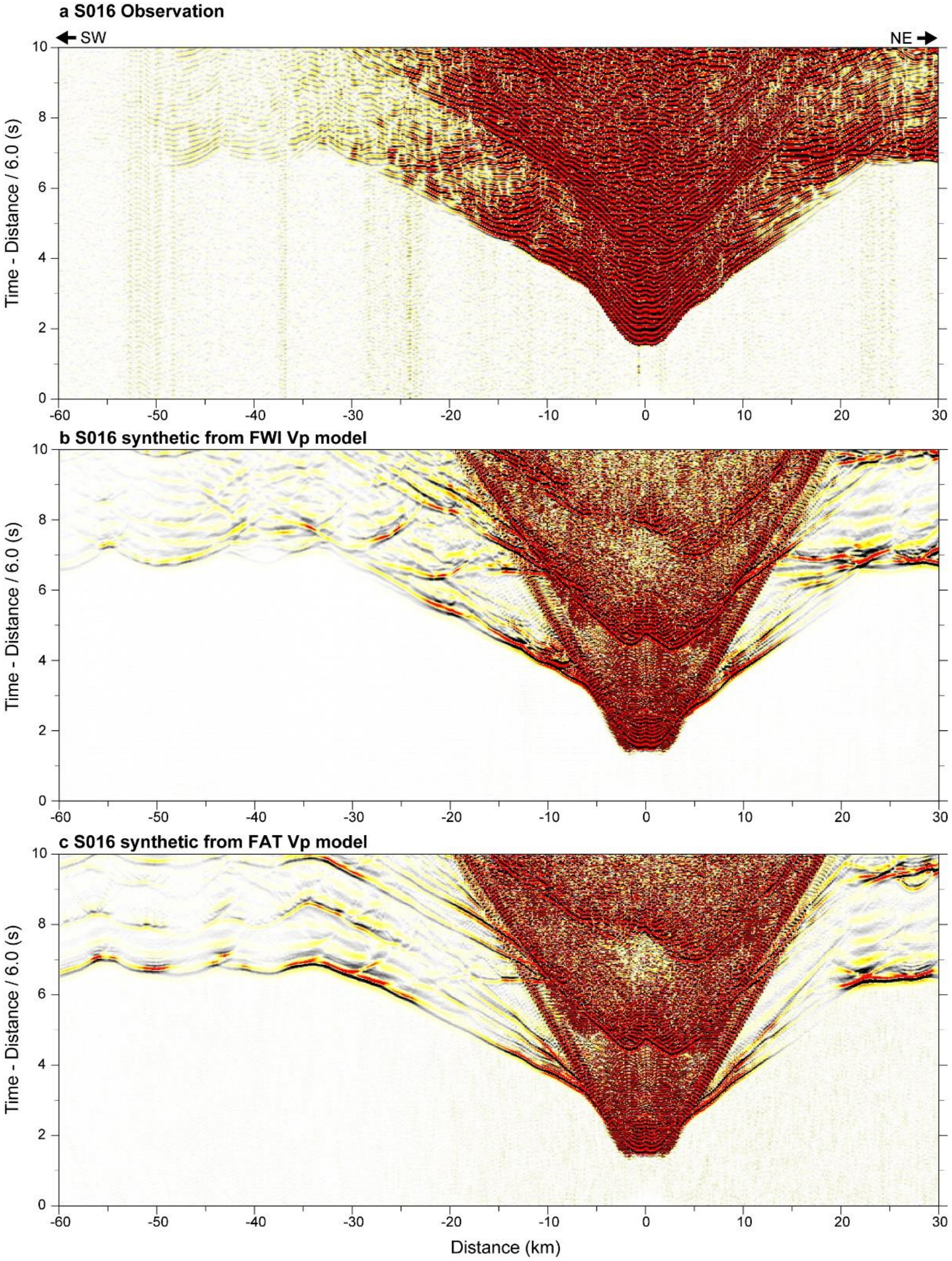

Supplementary Figure 6. Comparison of ocean bottom seismograph (OBS) records of S016 with synthetic waveforms. (a) Observed records of vertical geophone data. (b) Synthetic waveforms calculated from the final full waveform inversion (FWI) P-wave velocity (Vp) model (Supplementary Fig. 9c). (c) Synthetic waveforms calculated from the first-arrival traveltime tomography (FAT) Vp model (Supplementary Fig. 9b); we applied a 2–8-Hz band-pass filter.

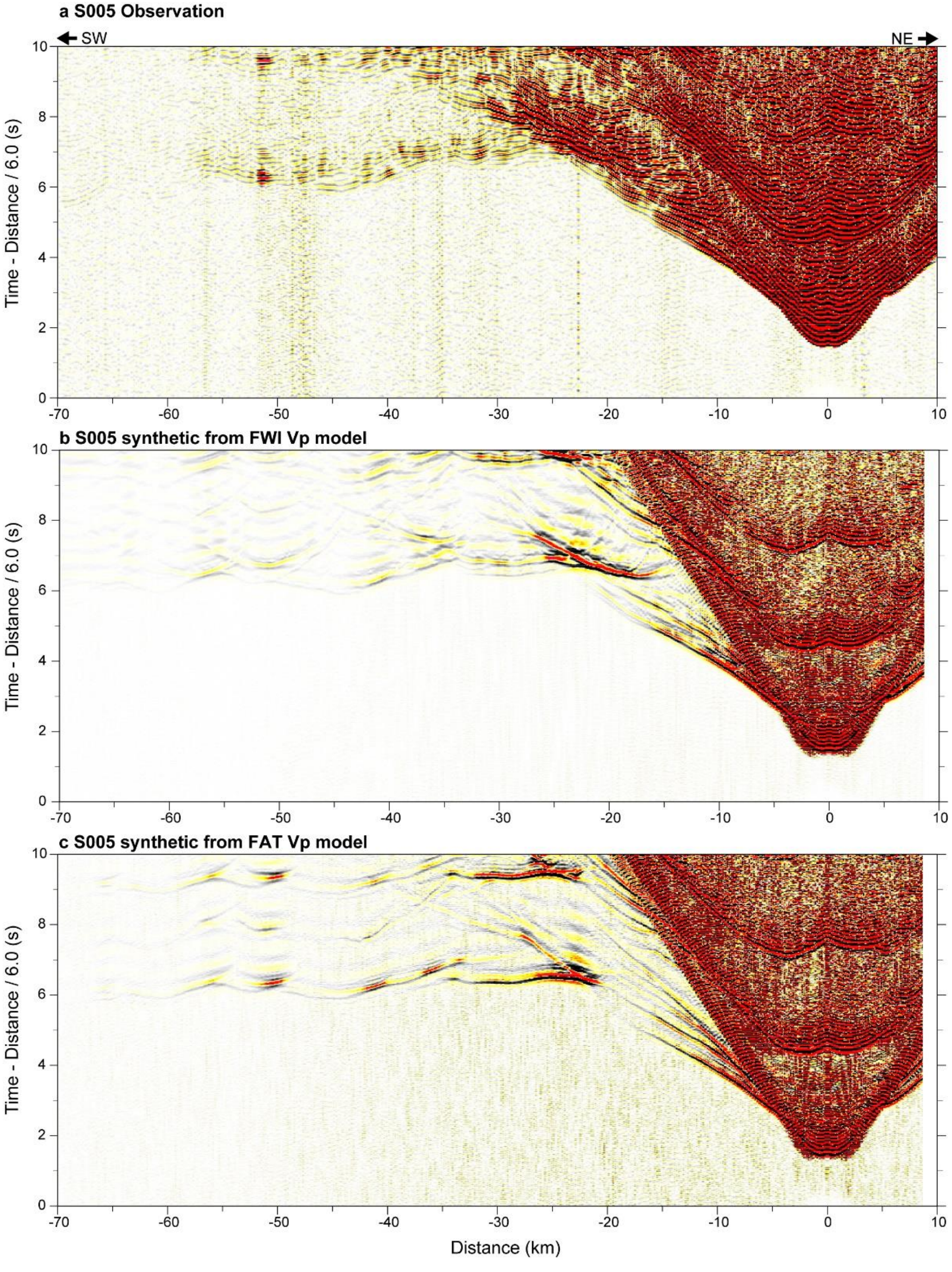

Supplementary Figure 7. Comparison of ocean bottom seismograph (OBS) records of S005 with synthetic waveforms. (a) Observed records of vertical geophone data. (b) Synthetic waveforms calculated from the final full waveform inversion (FWI) P-wave velocity (Vp) model (Supplementary Fig. 9c). (c) Synthetic waveforms calculated from the first-arrival traveltime tomography (FAT) Vp model (Supplementary Fig. 9b); we applied a 2–8-Hz band-pass filter.

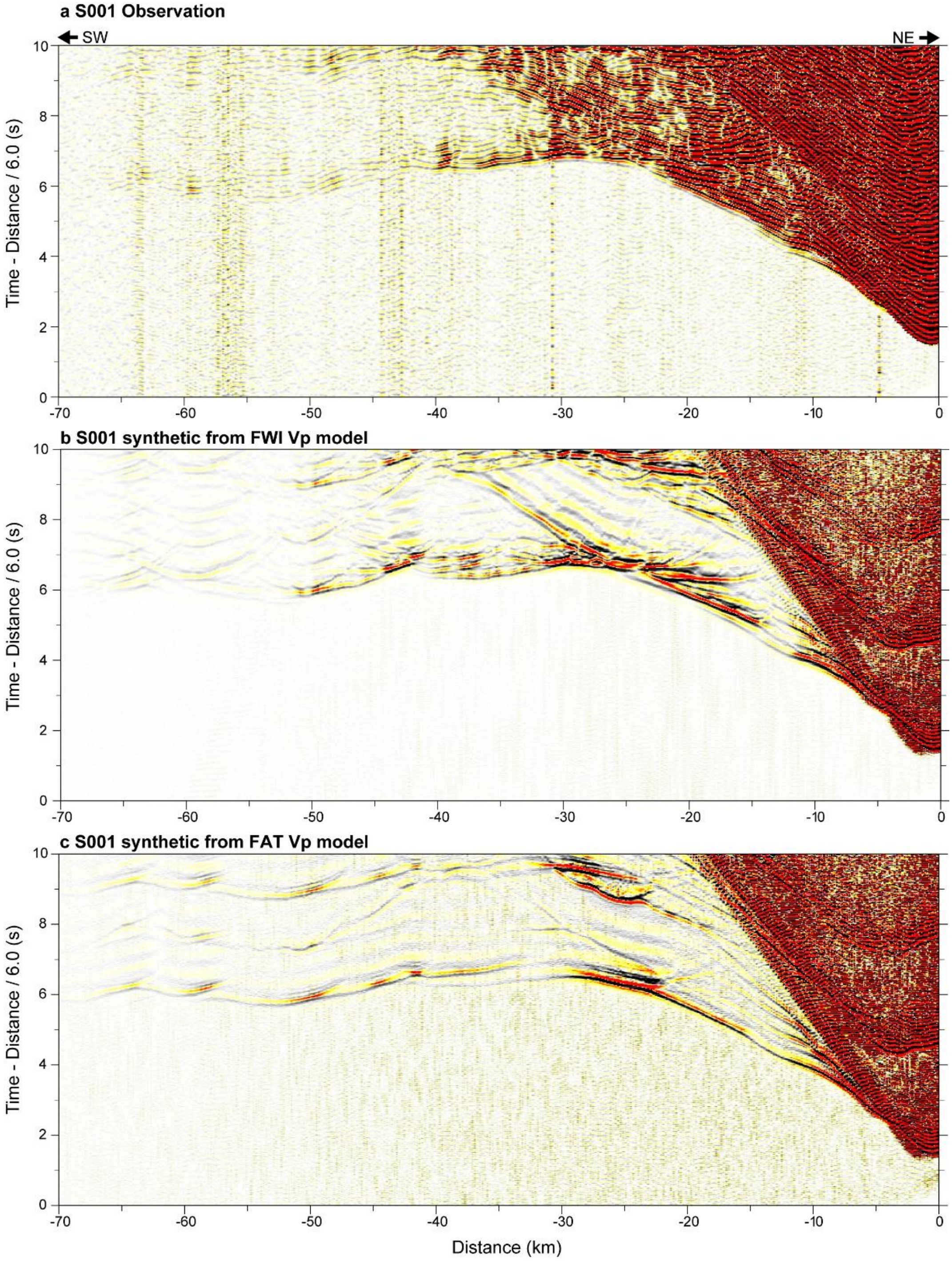

Supplementary Figure 8. Comparison of ocean bottom seismograph (OBS) records of S001 with synthetic waveforms. (a) Observed records of vertical geophone data. (b) Synthetic waveforms calculated from the final full waveform inversion (FWI) P-wave velocity (Vp) model (Supplementary Fig. 9c). (c) Synthetic waveforms calculated from the first-arrival traveltime tomography (FAT) Vp model (Supplementary Fig. 9b); we applied a 2–8-Hz band-pass filter.

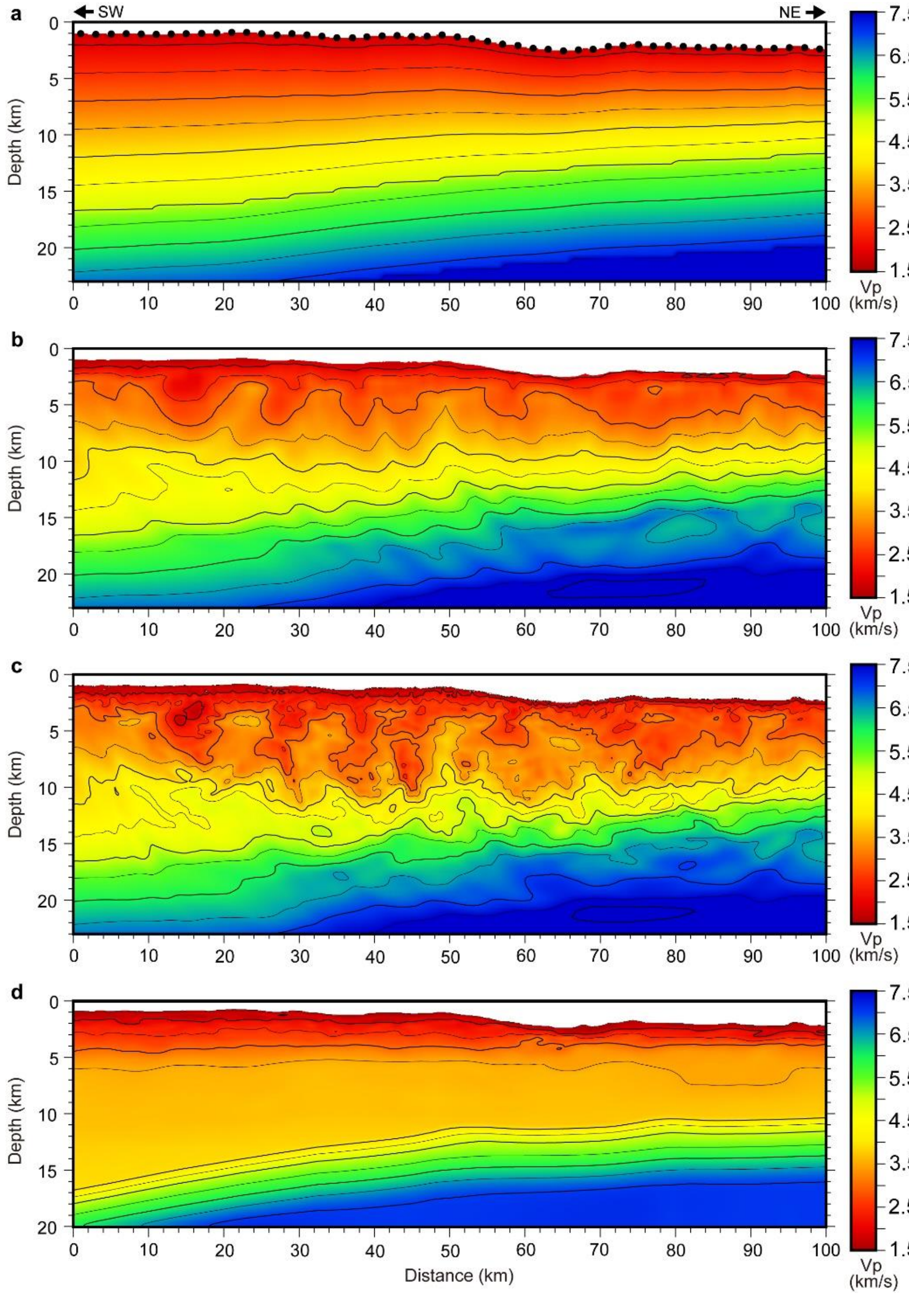

Supplementary Figure 9. Initial and final Vp modeling results. (a) Initial Vp model for the first-arrival traveltime tomography (FAT). The model was developed by referring to the Vp profiles from seismic refraction studies close to our survey line[1]. Black dots indicate the ocean bottom seismographs (OBS) locations. (b) Final Vp model by first arrival tomography. The root-mean-squared traveltime residual was reduced from 978 ms for the initial model to 56 ms after 15 iterations of inversion. This model was used as the starting model of FWI tomography. (c) Final Vp model from the FWI tomography. (d) Vp model from the pre-stack depth migration of muti-channel seismic reflection data.

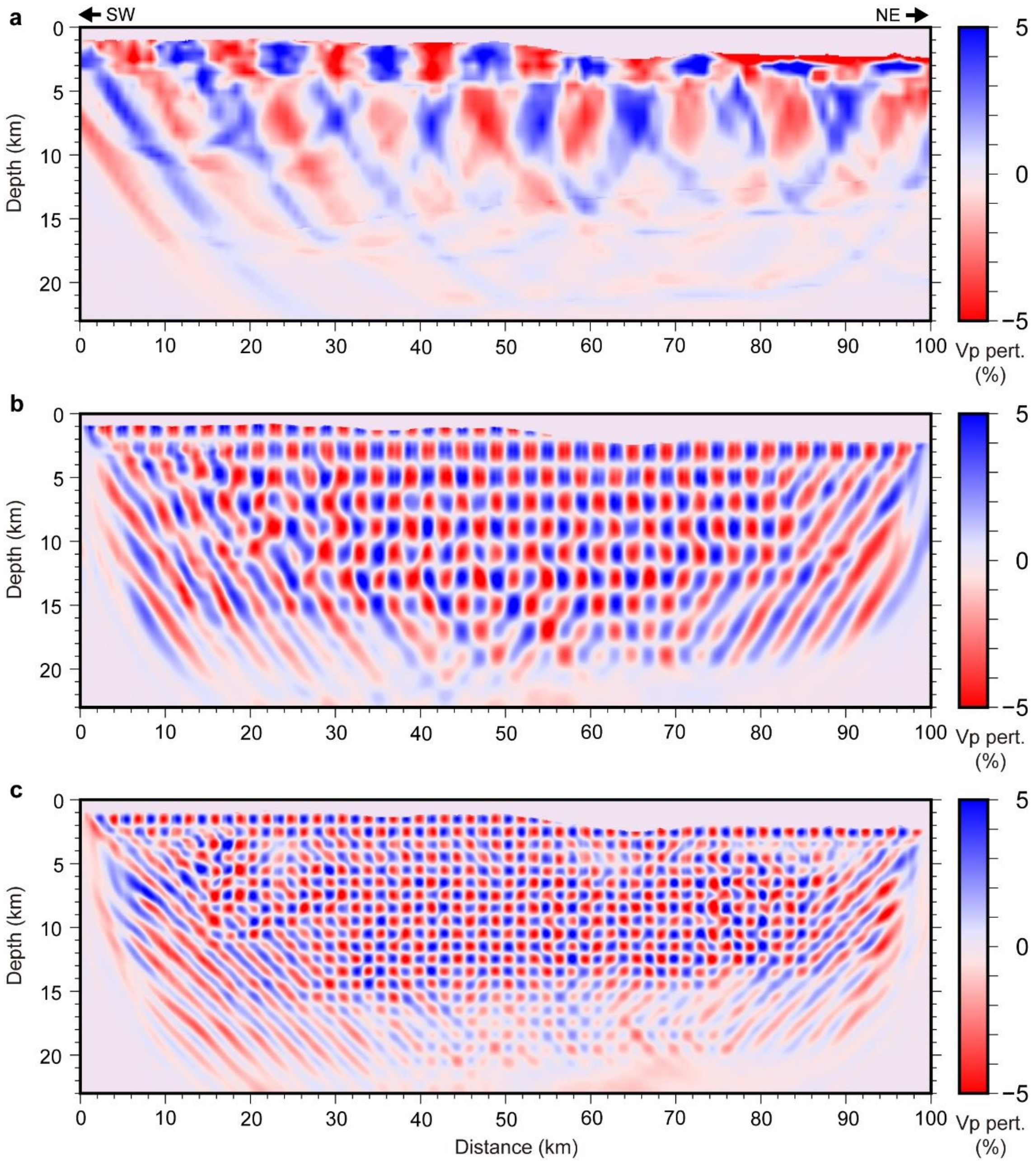

Supplementary Figure 10. Checkerboard resolution tests for first-arrival traveltime tomography (FAT) and full waveform inversion (FWI) tomography. (a) Result of FAT, with an anomaly size of 5 km horizontally x 4 km vertically. (b) Result of FWI, with an anomaly size of 2 km horizontally x 2 km vertically. (c) Result of FWI, with an anomaly size of 1.5 km horizontally x 1 km vertically. A reference model was created by adding sinusoidal anomalies, with a maximum relative amplitude of 5 %, to the final FAT model (Supplementary Fig. 9b).

Supplementary Table 1. FWI parameters. Each inversion step includes 20 iterations of inversion at maximum. Offset indicates the distance between shot and receiver within which the waveform data were used in the FWI analysis. Time damping indicates the parameter for the time window from the first arrival time (Supplementary Figure 11). Cost function reduction is the ratio of cost function between initial and final models at each inversion step. After the 25 inversion steps, the cost function reduced 94.5% from the initial FAT model.

Inversion step	Frequency (Hz)	Offset (km)	Time damping	Cost function reduction (%)
1	2.5, 2.75, 3.0, 3.25, 3.5	20	5.0	29.9
2	2.5, 2.75, 3.0, 3.25, 3.5	40	5.0	10.9
3	2.5, 2.75, 3.0, 3.25, 3.5	60	2.5	1.5
4	2.5, 2.75, 3.0, 3.25, 3.5	80	1.0	4.0
5	2.5, 2.75, 3.0, 3.25, 3.5	80	0.5	0.8
6	2.5, 2.75 … (every 0.25) … 4.25, 4.5	20	5.0	26.5
7	2.5, 2.75 … (every 0.25) … 4.25, 4.5	40	5.0	4.8
8	2.5, 2.75 … (every 0.25) … 4.25, 4.5	60	2.5	1.3
9	2.5, 2.75 … (every 0.25) … 4.25, 4.5	80	1.0	0.9
10	2.5, 2.75 … (every 0.25) … 4.25, 4.5	80	0.5	0.3
11	2.5, 2.75 … (every 0.25) … 5.25, 5.5	20	5.0	10.7
12	2.5, 2.75 … (every 0.25) … 5.25, 5.5	40	5.0	15.5
13	2.5, 2.75 … (every 0.25) … 5.25, 5.5	60	2.5	2.2
14	2.5, 2.75 … (every 0.25) … 5.25, 5.5	80	1.0	1.3
15	2.5, 2.75 … (every 0.25) … 5.25, 5.5	80	0.5	0.5
16	2.5, 2.75 … (every 0.25) … 6.25, 6.5	20	5.0	22.1
17	2.5, 2.75 … (every 0.25) … 6.25, 6.5	40	5.0	2.9
18	2.5, 2.75 … (every 0.25) … 6.25, 6.5	60	2.5	1.9
19	2.5, 2.75 … (every 0.25) … 6.25, 6.5	80	1.0	0.7
20	2.5, 2.75 … (every 0.25) … 6.25, 6.5	80	0.5	1.6
21	2.5, 2.75 … (every 0.25) … 7.25, 7.5	20	5.0	17.4
22	2.5, 2.75 … (every 0.25) … 7.25, 7.5	40	5.0	1.8
23	2.5, 2.75 … (every 0.25) … 7.25, 7.5	60	2.5	1.2
24	2.5, 2.75 … (every 0.25) … 7.25, 7.5	80	1.0	0.8
25	2.5, 2.75 … (every 0.25) … 7.25, 7.5	80	0.5	1.3

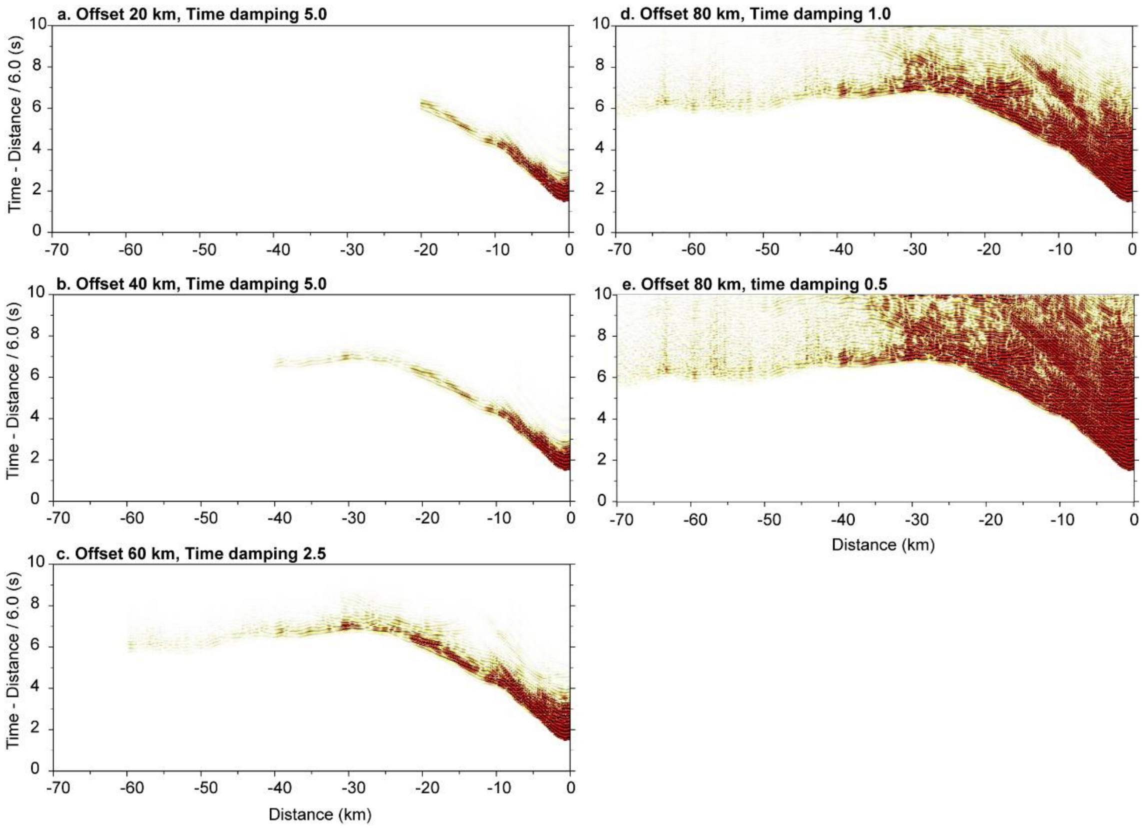

Supplementary Figure 11. Offset and time damping used in FWI analysis. (a) Waveform records with the offset of 20 km and the time damping of 5.0. (b) Waveform records with the offset of 40 km and the time damping of 5.0. (c) Waveform records with the offset of 60 km and the time damping of 2.5. (d) Waveform records with the offset of 80 km and the time damping of 1.0. (e) Waveform records with the offset of 80 km and the time damping of 0.5. The data are muted before the first arrival and the exponential time damping is applied after the first arrival. The five panels correspond to the parameters used in first to fifth steps at each frequency band (Supplementary Table 1).

Supplementary References

1. Nakanishi, A., Takahashi, N., Yamamoto, Y., Takahashi, T., Citak, S. O., Nakamura, T., Obana, K., Kodaira, S. & Kaneda, Y. Three-dimensional plate geometry and P-wave velocity models of the subduction zone in SW Japan: Implications for seismogenesis, in *Geology and Tectonics of Subduction Zones: A Tribute to Gaku Kimura*, 69–86, eds Byrne, T., Underwood, M.B., Fisher, D., McNeill, L., Saffer, D., Ujiie, K. & Yamaguchi, A. Geological Society of America Special Paper 534 (2018). doi:10.1130/2018.2534(04)
